# Guiding Principles for Transformation Towards Integrated Acute Care for Older Adults Close to Home: Lessons from Nine Dutch Regional Partnerships. A Realist Evaluation

**DOI:** 10.5334/ijic.8967

**Published:** 2025-07-08

**Authors:** Eline D. Kroeze, Gercora Hoitinga, Susanne M. Smorenburg, Janet L. MacNeil Vroomen, Anneke J. A. H. van Vught, Bianca M. Buurman

**Affiliations:** 1 Department of Internal Medicine, Section of Geriatric Medicine, Amsterdam UMC, location University of Amsterdam, Amsterdam, The Netherlands; 2 Amsterdam Public Health, Aging Later Life, Amsterdam, The Netherlands; 3 Department of Medicine for Older People, Amsterdam UMC, location Vrije Universiteit Amsterdam, Amsterdam, The Netherlands; 4 Amsterdam UMC location AMC, Department of Emergency Medicine, Amsterdam, The Netherlands; 5 Dutch Healthcare Authority (NZa), Utrecht, The Netherlands; 6 Canisius Wilhelmina Hospital, Nijmegen, The Netherlands

**Keywords:** integrated care, acute care, older people, intermediate care, realist evaluation, micro foundations

## Abstract

**Background:**

Integrated Acute Care for Older People (IACOP) close to home is a promising approach to overcome care fragmentation, but little is know about effective meso- and macro-strategies to foster integration. This Realist Evaluation (RE) explores which strategies were employed and recommended in nine Dutch regional partnerships.

**Methods:**

The Rainbow Model, RE-theory, Coleman’s boat and ripple effects functioned as theoretical basis. We conducted a document analysis (n = 19), semi-structured interviews (n = 11) and two focus groups (n = 8). Participants were (project) managers, directors and experts. The qualitative data were clustered into guiding principles, underpinned with strategy-context-mechanism-outcome configurations.

**Results:**

Six guiding principles for transformation towards IACOP close to home emerged. Meso-level principles included: 1) committing to a shared regional IACOP vision and goals; 2) fostering a culture of collaborative and coordinated action; 3) prioritising, implementing and developing micro-level interventions systematically. Macro-level principles were: 4) ensuring congruent policy; 5) stimulating functional integration and 6) stimulating normative integration.

**Conclusions:**

Transforming towards IACOP close to home is complex and requires continuous action across integration levels, health practices and sectors. The meso-level principles guide regional partnerships in applying context-specific strategies; activating the underlying mechanisms for transformation like ‘enjoying the process’. Yet, successful transformation also hinges on actions by system stakeholders.

## Introduction

Like many countries, the Netherlands encounters challenges in providing healthcare to its ageing population. Major reforms to the healthcare system were introduced in 2006 and 2015 to meet this trend and to control for growing costs [1,2]. Since 2015, there has been a shift in focus towards ‘ageing in place’, accompanied by stricter admission criteria for long-term care (LTC) with nursing homes only admitting persons needing continuous care [2]. Consequently, a growing number of older people ‘age in place’ and receive care at home from a range of professionals from diverse organisations, supported by varying financial systems and healthcare sectors [3]. Within this fragmented system, older people face elevated risks of emergency department visits and hospitalisations [4,5]. Once admitted to the hospital and/or during transitions from hospital to home, they are at increased risks of adverse health outcomes including loss of function, rehospitalisation and death [6,7,8].

Integrated Acute Care for Older People (IACOP) close to home is a promising approach to overcome acute care fragmentation for older adults living at home. The objective is to prevent acute care needs or to resolve them as close to home as possible, with the overarching ambition to achieve the Quadruple Aim [9]. Acute care encompasses all unexpectedly required care. Within IACOP, preventive, acute and post-acute care is person-centred, with the older person’s wishes and care needs as central focus. This requires attention to not only the medical problem(s), but also to functional capacity, performance status, comorbidities, polypharmacy, cognition, nutritional status, psychological status and social support [10]. Also, the principles of positive health [11], reablement [12] and shared-decision making are applied [13] and the older person experiences continuity of care, as care is coordinated among the involved healthcare professionals along the care continuum.

To foster transformation towards IACOP close to home, intermediate models have been developed and implemented in the Netherlands. These models integrate care, ensure continuity and quality of care, and promote recovery at the interface between hospital, home, care home, primary care and community services. They support coordinated action between professionals and organisations, are provided close to home, support early discharge and reduce (re)admissions to acute care [14,15]. Examples are generalist-specialist collaboration in primary care [16], the acute geriatric community hospital (AGCH) [17], short-term residential stay (STRC) [18] and outpatient geriatric rehabilitation (OGR) [19]. All focusing on preventing functional decline and frailty in older adults by targeting care integration at the micro-level.

While there has been an increasing focus on micro-level strategies for integrating care for older people, there is a knowledge gap regarding meso- and macro-level strategies. Briggs et al. advocated for more evidence on these to achieve implementation of integrated care at scale and highlighted the importance of linking outcomes to contexts [20]. With this Realist Evealution (RE), we responded to this call of action and monitored nine regional partnerships working towards IACOP close to home. These partnerships offered detailed insights into the meso-level strategies they applied and the macro-level strategies they recommended to foster transformation towards IACOP close to home. The aim of this article is to provide insight into which meso- and macro-strategies were used and recommended, by whom, why and when, based on the experiences of these regional partnerships. The following research question will be answered: Given the developments within nine Dutch regional partnerships, what are the guiding principles for regional partnerships and system stakeholders to facilitate transformation towards IACOP close to home?

## Theoretical framework

### The Rainbow Model of Integrated Care

**Figure 1:**
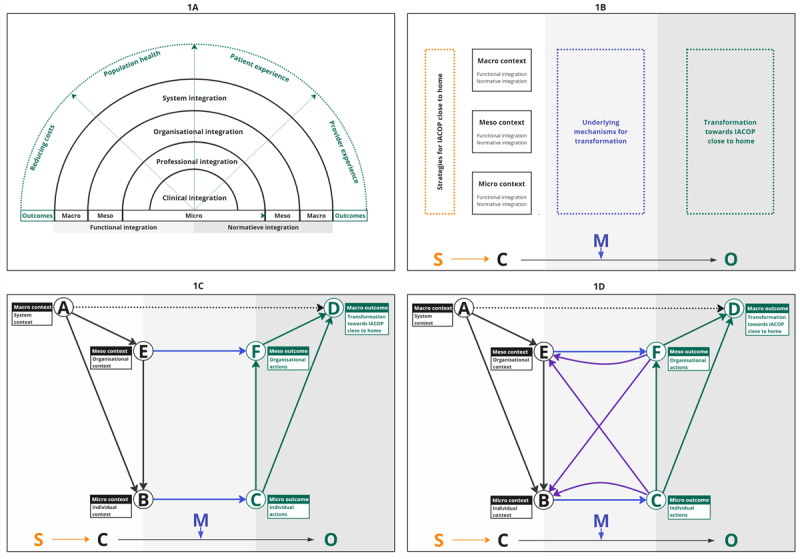
Step-by-step development of a conceptual model combining the Rainbow Model of Integrated Care [21] **(1A)**, Realist Evaluation theory [22,23] **(1B)**, Coleman’s boat [26,27] **(1C)** and ripple effects [28] **(1D)**. The abbreviations used are S, strategy; C, context; M, mechanism; O, outcome; IACOP, integrated acute care for older people. The solid black arrows (#AB, #AE, #EB) represent situational mechanisms; the blue arrows represent individual (#BC) and organisational (#EF) action formation mechanisms; the green arrows (#CD, #CF, #FD) represent transformational mechanisms; the purple arrows represent ripple effects (#CB & #FE & #CE & #FB) and the black dotted arrow (#AD) represents a simplified view of a pure macro relationship. The nodes depicted are A, the macro context (system context); B, micro context (individual context); C, micro-outcome (individual actions); D, macro-outcome (transformation towards IACOP close to home); E, meso context (organisational context); and F, meso outcome (organisational actions).

To understand integrated care and its multilayered nature, the Rainbow Model of Integrated Care (RMIC) was used (see Figure 1A). The RMIC describes the different types of integration aimed at the micro-, meso- and macro-level to achieve the Quadruple-Aim [21]. In this study, we placed individuals working on clinical and professional integration at the micro-level; organisations working together in regional partnerships at the meso-level; and system stakeholders at the macro-level. Context factors that enable or inhibit integration, can be functional (e.g. ‘hard’ factors like financing and communication systems) or normative (e.g. ‘soft’ factors like cultural values and vision).

### Combining the RMIC and RE theory

We applied a RE approach to gain insight into what works, for whom, in which context, when and why [22,23]. We identified different strategies (S), contexts (C) and mechanisms (M) that were triggered and generated transformation towards IACOP close to home (see Table 1). These SCMO configurations serve as heuristics to elucidate why a strategy is successful in context A, but not in context B. In Figure 1B, the RMIC and RE theory are combined into a conceptual model. Inherent to RE theory is the understanding that programs are complex and context-dependent, with outcomes arising from non-linear, unpredictable interactions between strategies, contexts, mechanisms, and outcomes [24,25].

**Table 1:** Definitions of main concepts of the SCMO configurations.


Strategy (S)	Refers to intended plans of action [23]. In this study, strategies are aimed at transforming towards IACOP close to home.

Context (C)	Conditions that trigger or modify the mechanism [23]. In this study the contextual factors are functional or normative and apply to the individual (micro), organisational (meso) or system level (macro).

Mechanism (M)	Refers to the generative force that leads to transformation [23]. Mechanisms are no plans of actions, but responses to the strategies applied in differed contexts: they explain why strategies work or not.

Outcome (O)	Refers to intended or unintended process outcomes [23]. In this study we defined outcomes as individual and/or organisational actions that either positively or negatively impact transformation towards IACOP close to home.


### Adding Coleman’s Boat

In line with the principles of the RMIC model, the underlying assumption of this study is that the system level (macro) influences transformation towards IACOP close to home through decision-making by individuals (micro) who work within stakeholder organisations (meso). To research these micro foundations, we incorporated the elements of Coleman’s Boat [26] into our conceptual model (see Figure 1C). Micro foundations refer in this study to the behaviour, decisions, or (inter)actions of individuals and organisations that collectively contribute to transformation towards IACOP close to home.

The nodes on the upper level and arrow(#) AD represent a simplified view of a pure macro relationship, where the macro context contributes to the macro-outcome. The nodes on the lower level and #BC represent a simplified view of a micro relationship, where the micro context (individual context) shapes micro outcomes (individual actions) through ‘individual action formation mechanisms’. The nodes on the middle level illustrate a meso relationship, where the meso context (organisational context) influences meso outcomes (organisational actions) through ‘organisational action formation mechanisms’. When individuals and/or organisations are situated in varying contexts, different underlying mechanisms are triggered, influencing how they will act.

The model acknowledges that the macro outcome emerges from the aggregation of individual actions (#CD) and organisational actions (#FD) through ‘transformational mechanisms’. Furthermore, individual actions are not only directly related to macro outcome(s), but also indirectly by influencing organisational actions (#CD). The remaining arrows connect the three-level contexts through ‘situational mechanisms’. The macro-to-meso-level is connected by #AE, the meso-to-micro-level by #EB and the macro-to-micro-level by #AB [27].

### Adding ripple effects

To account for non-linearity in our model, we incorporated ripple effects as feedback loops [28], demonstrating how the outcome(s) of a SCMO configuration may become (an aspect of) the context in another configuration (see Figure 1D). For example, a micro outcome, such as more encounters between two individuals (node C), can become (an aspect of) a new context (node B), where increased mutual understanding (#BC) leads to (more) effective collaboration (node C). The meso-level ripple effects are represented by #FE. Finally, individual actions may alter the meso context through the ripple effect #CE and organisational actions may alter the micro context through ripple effect #FB. The final model consists of three levels, two nodes at every level, and causal arrows between these nodes.

## Methods

### Policy context

The nine regional partnerships (see Table 2 and appendix 1) operate in the Dutch healthcare system, which is broadly based on three principles: 1) universal access to healthcare while allowing “regulated competition” between care providers, 2) solidarity through medical insurance which is required for all residents, and 3) high-quality healthcare services [29]. Three of the four basic care-related acts that govern the healthcare system apply to care for older people in the Netherlands. More information on these acts is provided in appendix 1.

**Table 2:** Regional partnerships for IACOP close to home.


	DEGREE OF URBANISATION [32]	PARTNERS FOR IACOP CLOSE TO HOME

**R1**	Hardly urbanised	1 general hospital, 1 nursing (home) organisation, 1 GP care group, 1 health insurer

**R2**	Moderately urbanised	1 general hospital, 1 nursing (home) organisation, 1 health insurer

**R3**	Hardly urbanised	1 topclinical hospital, 1 nursing (home) organisation, 1 health insurer

**R4**	Moderately urbanised	1 topclinical hospital, 1 nursing (home) organisation, 1 GP care group, 2 health insurers

**R5**	Strongly urbanised	1 general hospital, 1 nursing (home) organisation, 1 GP care group, 2 health insurers

**R6**	Moderately urbanised	1 general hospital, 3 nursing (home) organisations, 3 GP care groups, 1 mental health organisation, 1 health insurer

**R7**	Moderately urbanised	1 topclinical hospital, 3 nursing (home) organisations, 1 GP care group, 1 health insurer

**R8**	Strongly urbanised	1 general hospital, 1 nursing (home) organisation, 1 GP care group, 1 health insurer

**R9**	Extremely urbanised	1 university hospital, 1 nursing (home) organisation, 1 GP care group, 1 health insurer


### Study design

We conducted an explorative qualitative Realist Evaluation (RE). We applied the COREQ-checklist [30] and RAMESES II reporting standards for REs [31] to ensure all items relevant to reporting qualitative REs were included (see appendix 2). The study protocol was submitted to the Amsterdam University Medical Centre’s Medical Ethics Research Committee (file number 2023.0478). The need for official approval was waived as the Medical Research Involving Human Subjects Act did not apply. Written consent was obtained from the participants.

### Participants and research team

The participants (n = 44), primarily project managers and directors, were involved in the implementation of intermediate care models for IACOP close to home, with a predominant focus on the AGCH. We did not include older adults, informal caregivers, and healthcare professionals as participants, as our aim was to get insight into meso- and macro-level strategies implemented and recommended by stakeholders within regional partnerships. Participants were involved in nine Dutch regional partnerships, spanning diverse contexts, including both rural and urban areas (see Table 2). They were employed by hospitals and/or nursing (home) organisations. A purposive sampling method was used to obtain interview participants from each regional partnership. Participants were recruited via an e-mail explaining the goal of the study. The research team consisted of a PhD student with a health economic background (EK, MSc), a PhD student with a nursing background (GH, MSc), the AGCH program manager (SS, PhD), an associate professor with expertise on ‘ageing in place’ policies (JM, PhD), an associate professor with expertise on ‘the right care at the right place’ (AV, PhD), and a professor in acute geriatric care (BB, PhD).

### Data collection

The data for this study was gathered from November 2021 to December 2023. The research was based on stakeholders’ experiences identified in 1) minutes of meetings between directors and project managers of nine different regional partnerships, in 2) semi-structured interviews with project managers and in 3) focus groups with project managers and experts (see Table 3).

**Table 3:** Data collection and study participants.


TYPE OF DATA	DESCRIPTION	PARTICIPANTS	OCCUPATION	ACTIVE IN REGION (R)

Documents	Minutes of 7 director meetings	19	Directors	1×R1, 3×R2, 2×R3, 3×R4, 4×R5, 2×R6, 1×R7, 2×R8, 1×R9

Documents	Minutes of 12 project leader meetings	22	Project managers	3×R1, 2×R2, 1×R3, 1×R4, 5×R5, 2×R6, 2×R7, 4×R8, 2×R9

Interviews	Transcripts of 10 semi-structured interviews	11	Project managers	2×R1, 2×R2, 1×R3, 1×R4, 2×R5, 1×R6, 1×R7, 1×R8

Focus groups	Transcripts of 2 focus groups	8	5 project managers 3 experts	1×R1, 1×R2, 1×R4, 1×R5, 1×R7 –


#### Documents

Between May 2022 and October 2023, seven one-hour meetings were organised for directors of stakeholder organisations from the nine regional partnerships. Also, 12 one-hour meetings were organised for the project managers of the nine regional partnerships between 2021 and April 2023. SS chaired the meetings and EK wrote detailed minutes. In total 19 meeting minutes were gathered.

#### Semi-structured interviews

One or two project managers per region, excluding region nine due to the lack of a formal project manager, were interviewed on their experiences with transforming towards IACOP close to home. First, the regional context and micro-level strategies applied along the acute care continuum for older adults were discussed. Then, we delved into meso- and macro-level strategies and contextual factors that facilitate or hinder transformation, utilising the RMIC framework as guidance. The average duration of the interviews was 80 minutes each.

#### Focus groups

While the document analysis and semi-structured interviews provided much information on the strategies and contexts for IACOP close to home, two focus groups with project managers (n = 5) and IACOP experts (n = 3) were held to explicitly define the underlying mechanisms for transformation. Based on the data from the document analysis and semi-structured interviews, possible mechanisms for transformation were proposed, discussed and refined. Also, the focus group members member-checked the guiding principles for transformation and the underlying SCMO configurations. Each focus group lasted 90 minutes.

### Data analysis

The analysis of the documents, semi-structured interviews and focus groups can be divided into three iterative steps.

#### Identification of SCMO configurations

The semi-structured interviews were transcribed verbatim and, together with the (anonymised) meeting minutes, analysed thematically [33] in MAXQDA 2022 by EK using both a deductive and inductive approach. A priori SCMO categories were drawn from RMIC and RE theory (see Figure 1B). GH also coded one interview and two minutes of meetings. GH and EK held reflexive meetings to establish coding accuracy and confirmability of findings.

#### Clustering the SCMO configurations into guiding principles

The identified SCMO configurations were merged into larger overarching configurations and thematically clustered into guiding principles by EK and cross-checked by the research team (GH, AV, SS, BB). In total nine guiding principles were constructed.

#### Refinement of the SCMO configuration and guiding principles

Based on the feedback of focus group participants, the SCMO configurations were revised. Also, the researchers tried to gain more understanding on the relations between the micro-meso-macro contexts and outcomes grounded in the RMIC [21], RE-theory [22,23] Coleman’s boat [26,27] and ripple effects [28]. EK and AV refined and rearranged the SCMO configurations during an iterative process an regrouped them into six guiding principles. These were reviewed and confirmed by GH, SS and BB.

## Results

**Figure 2:**
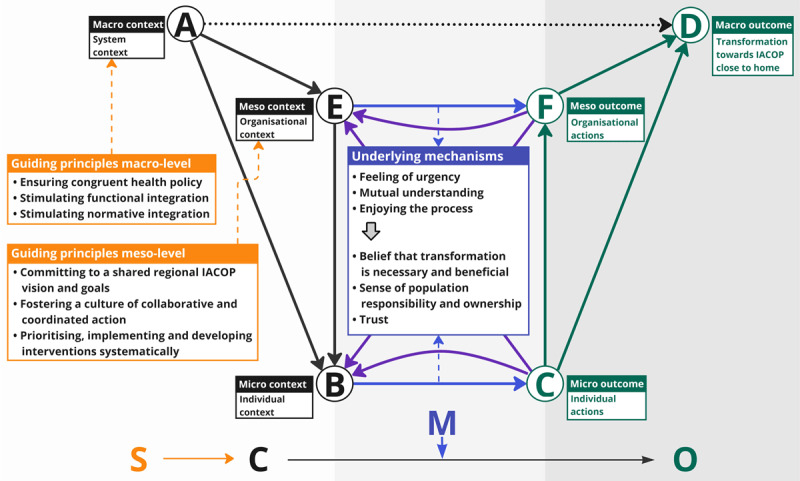
Findings summarised into the conceptual model. The abbreviations used are S, strategy; C, context; M, mechanism; O, outcome; IACOP, integrated acute care for older people. The solid black arrows (#AB, #AE, #EB) represent situational mechanisms; the blue arrows represent individual (#BC) and organisational (#EF) action formation mechanisms; the green arrows (#CD, #CF, #FD) represent transformational mechanisms; the purple arrows represent ripple effects (#CB & #FE & #CE & #FB) and the black dotted arrow (#AD) represents a simplified view of a pure macro relationship. The nodes depicted are A, the macro context (system context); B, micro context (individual context); C, micro-outcome (individual actions); D, macro-outcome (transformation towards IACOP close to home); E, meso context (organisational context); and F, meso outcome (organisational actions).

The SCMO configurations were grouped into main themes of guiding principles (see Figure 2), which are considered to positively influence actions of individuals and organisations for regional transformation towards IACOP close to home. Three meso-level guiding principles were formulated for regional partnerships: 1) committing to a shared regional IACOP vision and goals; 2) fostering a culture of collaborative and coordinated action; and 3) prioritising, implementing and developing interventions systematically. Also, three macro-level guiding principles were formulated for system stakeholders: 4) ensuring congruent health policy; 5) stimulating functional integration; and 6) stimulating normative integration. Three primary mechanisms were identified: 1) a feeling of urgency, 2) mutual understanding and 3) enjoying the process. Once initiated, these mechanisms activated the three secondary mechanisms: 4) a belief that transformation is necessary and beneficial, 5) a sense of population responsibility and ownership, and 6) trust. Successfully activating the underlying mechanisms for transformation depended on applying the right strategies in the right contexts at the right time. The identified contextual factors across the micro-, meso- and macro-levels concerned the distribution of care, the communication structure, funding, vision and culture (see appendix 3).

### Three meso-level guiding principles

In this section, we describe each guiding principle for regional partnerships followed by example strategies that were implemented and how, when and why these strategies did (or did not) contribute to transformation in different contexts.

#### Committing to a shared IACOP vision and goals

When working within a region with multiple different stakeholders for IACOP close to home the participants addressed the importance of shared regional IACOP vision and goals:

“*‘What is our success story?’ That is that we formulated a regional vision that is acknowledged by all regional stakeholders*.” – Respondent B, interview 14/03/23.

To create and maintain commitment to the regional IACOP vision and goals, two types of strategies were applied: 1) a shared vision and IACOP goals were created in line with urgent issues that resonated with patients and (healthcare) professionals and 2) leaders continuously communicated the shared IACOP vision and goals.

The second strategy varied in success, depending on the mechanisms that were triggered in various contexts. For example, continuous communication on the shared IACOP vision and goals (S) was more challenging in organisations with (unforeseen) changes in board and/or management (C). A lack of mutual understanding between healthcare staff and the new board member(s) and/or manager(s) (M), combined with inconsistent communication of the regional vision and goals (C), contributed to damaged trust in the transformation process (M), resulting in less individual contribution to transformation (O).

#### Fostering a culture of collaborative and coordinated action

“*There is nothing quite as complex as changing culture*.” – Respondent D, interview 17/04/23

Though culture change is complex, participants agreed that the time has passed to see each other as competitors; collaborative and coordinated action is required to achieve IACOP close to home.

Applied strategies at the professional level encouraged more frequent interactions between (health)care professionals from different sectors and practices. At the organisational level, stakeholders invested in strategies to (re)structure the regional partnership’s governance.

The participants observed that there are often distinct work cultures between organisations and professionals from different practices and/or sectors (C). Frequently, (healthcare) professionals lack adequate understanding of each other’s practice (C) and may not communicate using the same terminology (C). Therefore, regional stakeholders promoted more frequent interactions (S) through co-locating their workspaces, by offering joint training sessions and internships or by introducing intermediate care models (like the AGCH) where an interdisciplinary team operates. When (healthcare) professionals from different healthcare practices and sectors interacted more frequently (C), mutual understanding increased (M). By using the ripple effect concept, this increased mutual understanding became a new context which triggered other mechanisms. When deeper mutual understanding allowed (healthcare) professionals to comprehend each other’s interests (C) and strengths and weaknesses (C), it fostered trust (M) among them and nourished the belief that transformation is both necessary and beneficial (M), ultimately advancing IACOP close to home (O).

#### Prioritising, implementing and developing micro-level interventions systematically

Participants noted that many projects for IACOP close to home were being initiated:

“*We started with a thousand blooming flowers; meaning that we gave a lot of initiatives a chance. This in the hope that some would become good examples with positive results, so that the ball would start rolling. But initiating that many projects did not lead to the desired outcome. Therefore, we decided to have more focus: currently we aim for fewer project, but the ones undertaken will have better support, progress, completion and results*.” – Respondent F, interview 23/04/23

Regional partnerships established more focus by 1) identifying shared bottlenecks for organising IACOP close to home, then 2) micro-level interventions were prioritised based on their expected impact. Subsequently, micro-level interventions were 3) systematically implemented and embedded in the regional healthcare system through continuous learning and development.

In one region, priority was given to micro-level interventions that alleviated the pressure on professionals in primary and district care (S). This prioritisation was chosen because older adults live longer at home (C) and the responsibility for basic health care lies with the GP and the district nurse (C). With the prospects of an ageing population (C), increasing multimorbidity (C), a tightening health labour market (C) and more hospital care shifting towards primary care (C), participants worried that the workload of these professionals would become too great (C). Even though GPs and district nurses may experience urgency (M) and a sense of population responsibility and ownership (M), they might lack the (mental) capacity (C) to enjoy (M) contributing to IACOP close to home (O).

Regional stakeholders applied different strategies to systematically implement micro-level interventions. For instance, two hospitals appointed medical managers in charge of transmural care (S) who have responsibility and ownership (M) for their hospital’s contribution to IACOP close to home (O). This triggered individual action (O) to reach organisational outcomes (O):

“*This person has a significant interest in the success of innovative interventions. Therefore, he/she usually offers their patients first, allowing us to make real progress. That’s very nice*.” – Respondent E, interview 31/05/23

Regional stakeholders embedded micro-level interventions in the regional healthcare system through continuous learning and development at the professional and organisational level. One example of the latter is that managers from care organisations and hospitals scheduled meetings to address joint capacity management:

“*I have regular meetings with managers of the hospital, long-term care organisations and the care coordination centre. We identify collaboratively who is responsible for client cases that are not properly referred. Subsequently, we work on how we can improve upon this*.” – Respondent B, interview 14/03/23

To implement effective joint capacity management at the organisational level (S), the participants emphasised the importance of a culture wherein individuals and organisations share their interests (C) and proceed common interests above personal and/or organisational interests (C). This culture can only exist if there is sufficient trust (M) between stakeholders that disclosing interests does not jeopardize the financial viability of one’s organisation (i.e. a ripple effect).

### Three macro-level guiding principles

In this section, we describe each guiding principle for system-level stakeholders followed by strategies that were recommended and how, when and why these strategies would contribute to transformation in different contexts.

#### Ensuring congruent health policy

The participants underlined the importance of health policy congruence. They recommended three strategies to ensure more congruent policy within and between system-level stakeholders: 1) allocating decisive roles, 2) committing to congruent behaviour, 3) communicating developments from management to operational staff (such as information on innovative care models, the regional contexts and health policy developments) and 4) providing generic care models open to context-based customisation.

When healthcare purchasers lack substantive knowledge about healthcare content (C) and/or when there is high turnover among purchasers (C), then the participants doubted whether the third strategy would promote enough mutual understanding (M) between healthcare providers and purchasers. Based on the experiences of the nine regional partnerships, this lacking mutual understanding became a new context (i.e. a ripple effect) wherein care providers engaged in numerous procurement meetings with health purchasers (C). If the individuals involved lost joy (M) and trust (M) in a positive and rapid outcome, their contributions to transformation diminished (O):

“*During procurement meetings, we had to answer the same questions repeatedly, causing the starting level at each meeting to drop to zero. Consequently, we have been facing financing challenges for more than a year. Therefore, it is crucial that healthcare insurers effectively communicate with their operational staff. Otherwise, we cannot maintain joy and sustain the innovation process*.” – Respondent Q, directors meeting 31/03/2023

#### Stimulating functional integration

The participants proposed several strategies for system-level stakeholders to coordinate and link financial, information and management systems around the process of integrated service delivery. These were categorised into; 1) aligning financial incentives with overarching system goals, 2) creating systems for regional care coordination and (patient) data exchange, 3) providing legal and quality frameworks for integrated care and 4) applying a flexible approach for functional integration.

Participants perceived the siloed reimbursement system (C) as a major barrier for realising IACOP close to home (O). Therefore, they advocated for integrated payment forms (S) like intersectoral payment categories, bundled payments or population-based payment. Yet, in a context where the healthcare system continuously changes (C), participants advocated for a flexible approach during financial integration (S):

“*… you cannot create change when the procurement frameworks are rigid. If the healthcare (system) changes, then the conditions for funding should also change*.”

– Respondent E, directors meeting 31/05/23

Owing to the rigid procurement frameworks (C), professionals engaged in the procurement of innovative care models needed to have considerable creativity and perseverance (C). The extensive effort and time invested, diminished joy (M) and trust (M) in de process, ultimately hampering (effective) individual actions for transformation towards IACOP close to home (O).

#### Stimulating normative integration

The participants indicated that policy documents from the government and other system-level stakeholders (S) provided organisations and individuals with clear perspectives on the future of the acute healthcare landscape. They noticed, however, that the role of the nursing (home) care sector is underexposed (C):

“*System-level stakeholders should realise that nursing (home) care also provides solutions for acute care*.” – Respondent L, directors meeting 29/06/23

Next to 1) formulating the role of nursing (home) care in national policy (visions), the participants recommended 2) managing citizens’ expectations about future care and 3) reframing and destigmatising ageing:

“*Older citizens are usually not excited to participate in a fall prevention program. They often see it as a sign of weakness. When, in fact, it should be reframed positively as ‘I am proactively taking action to stimulate self-reliance and to understand how to fall or how to catch myself when something like that happens’*.” – Respondent F, interview 23/04/23

If older adults experience urgency (M) and also understand (M) the importance of proactive action for self-reliance, then participants anticipated that more older individuals will undertake actions that facilitate transformation towards IACOP close to home (O).

## Discussion

Based on the experiences of nine Dutch regional partnerships, this study provided six guiding principles for transformation towards IACOP close to home. Three focusing on the meso-level: 1) committing to a shared regional IACOP vision and goals; 2) fostering a culture of collaborative and coordinated action; 3) prioritising, implementing and developing micro-level interventions systematically. The rest focusing on the macro-level: 4) ensuring congruent policy; 5) stimulating functional integration and 6) stimulating normative integration. We conclude that focusing on strategies which trigger the underlying mechanisms ‘a feeling of urgency’, ‘mutual understanding’ and ‘enjoying the process’ are most relevant when starting transformation. Once these mechanisms are activated, the mechanisms ‘a belief that transformation is necessary and beneficial, ‘a sense of population responsibility and ownership’ and ‘trust’ can be set in motion.

With this study we have contributed to narrowing the knowledge gap on effective meso- and macro-level strategies for regional integrated care. We emphasise that we did not identify linear cause-effect relationships between strategies, contexts, and outcomes (i.e. ‘attribution’), but acknowledge that other (contextual) factors may also have contributed to influencing outcomes (i.e. ‘contribution’). The formulated principles are in line with the CAHN components [34] and leading principles for Population Health Management [35]. Like van Vooren et al. [35], we identified the importance of shared commitment for a vision; mutual understanding and trust; responsibility; aligning policy; financial incentives; a learning cycle and stakeholder representation and leadership. Our research also confirms the importance of attending to relational dynamics when transforming towards IACOP close to home. In accordance with Gulati [36] we found that familiarity breeds trust between partners. This is founded in a common vision, based on mutually agreed goals, which in turn becomes the shared currency in a culture of collaborative and coordinated action. Moreover, our results highlight how contextual factors, such as regular interactions among (healthcare) professionals across different practices and sectors, provoke collaborative and coordinated action for IACOP close to home by (further) propelling the mechanisms ‘mutual understanding’, ‘trust’ and ‘the belief that transformation is both necessary and beneficial’.

Whilst we identified macro-level strategies for managing (older) citizens’ expectations about (future) care and reframing and destigmatising ageing, meso-level strategies concerning the active engagement of older citizens during transformation towards IACOP close to home were absent. This is noteworthy, considering the importance of older people’s participation in care integration to ensure that ‘the needs of the target population’ are reflected [37]. The absence may stem from not specifically addressing this subject during the interviews (i.e. information bias). It is also plausible that directors and program managers are insufficiently aware of the importance of involving older people as partners in transformation towards IACOP close to home. The latter would confirm a critical reflection by Glimmerveen et al. [37] that integrated care has become ‘too much a professional concept’ being pursued for (older) citizens, while insufficiently acknowledging the potential contribution made by (older) citizens.

Transforming health systems, as highlighted by Hunter and Bengoa, is a complex and messy process that requires both time and patience [38]. We also found that the secret lies in slow but consistent change, that is sustained through the ongoing application of context-specific strategies. In line with complex-adaptive systems thinking, we learned that regional health systems evolve when conditions are favourable [39]. Rather than being imposed, change emerges organically within an environment that fosters connectivity among professionals and organisations. Through ongoing interactions, creative solutions arise, driven by collective insights, shared decision-making, and continuous learning [40,41]. Barnsley et al. [42] add that facilitative leadership supports this process by removing obstacles and establishing open communication channels, allowing professionals and organizations to determine how to best achieve system goals.

To offer facilitative leaders in regional partnerships guidance on transforming towards IACOP close to home, we have incorporated our findings into Kotter’s framework for Leading Change [43] (see appendix 4). We recommend to start by creating a climate for transformation: 1) create a sense of urgency and 2) build a powerful guiding coalition. Then focus on commitment to a shared regional IACOP vision: 3) develop a vision with measurable goals and 4) continuously communicate these to maintain urgency levels. Then, 5) remove barriers to collaboration, joy and trust throughout the transformation process. Concurrently and/or subsequently, micro-level interventions must be prioritised, implemented and systemically developed: 6) start with quick wins to build momentum, then 7) sustain momentum by launching interventions that build on initial succesess. Finally, 8) anchor change by fostering a culture of continous learning and development for IACOP close to home.

To correct for information bias, the guiding principles and underlying SCMO configurations were member checked during the focus groups. We also tested for coding accuracy by carrying out spot checks. Table A5.1 in appendix 5 details the strategies applied to establish trustworthiness and rigour of our findings, categorised according to Lincoln and Guba’s four-dimension criteria framework [44]. All participants were actively involved in a Learning Network Acute Care for Older People. Since SS, AV and EK were coordinators of this Learning Network, they already had prior relationships with most participants. We believe that these prior relations did not threaten our study and actually encouraged participants to share their experiences and recommendations. By applying a RE-approach we increased the relevance and transferability of our findings; the SCMO configurations provide generalisable representations of which strategies work, for whom, when and in what circumstances [22,23]. The overarching guiding principles and mechanisms for transformation are transferable to other regional and national contexts. Due to the extensive and explorative nature of our study, we refrain from claiming data saturation. Furthermore, the breadth of our research prompted us to categorise the outcomes in our SCMO configurations as either positively or negatively impacting transformation towards IACOP close to home. This facilitated a deeper understanding of contexts, mechanisms and ripple effects. From a practical perspective, this deeper understanding is crucial to lead transformation towards IACOP close to home by applying context-specific strategies at the right time and in an effective sequence. From a scientific perspective, this deeper understanding is needed to accurately interpret integrated care outcomes across (regional) settings.

Our conceptual model can serve as theoretical foundation in further research on regional integrated care and can be applied to other complex adaptive systems. Continued monitoring of the Dutch regional partnerships for IACOP close to home would be valuable for linking the Quadruple [9] – or Quintuple [45] or Sextuple [46] – outcomes with strategies, contexts and mechanisms, and to determine whether additional SCMO configurations apply for regions in more advanced stages of transformation. We also recommend that future studies explore the experiences, expectations and recommendations of older adults, informal caregivers and healthcare professionals regarding IACOP close to home. Incorporating their perspectives would enhance person-centred care and drive accountability among stakeholders for IACOP close to home [47].

## Conclusions

Transforming towards IACOP close to home is complex and requires ongoing action across integration levels, healthcare practices and sectors. Three meso-level principles offer guidance for regional partnerships in applying context-specific strategies, activating underlying mechanisms such as mutual understanding and enjoying the process. Yet, successful transformation also requires system stakeholders to foster congruent policy, functional integration and normative integration. The 8-step process for leading transformation towards IACOP close to home may help regional partnerships and system stakeholders in deploying coordinated strategies across all levels of integration. Our conceptual model can be used as theoretical foundation in further research, enabling a deep understanding of contexts and mechanisms. This is crucial for accurately interpreting integrated care outcomes across various settings.

## Additional files

The additional files for this article are as follows:

10.5334/ijic.8967.s1 Appendix 1. Nine regional partnerships operating in the Dutch healthcare system.

10.5334/ijic.8967.s2 Appendix 2. RAMESES II and COREQ checklists.

10.5334/ijic.8967.s3 Appendix 3. The program theory and coding tree.

10.5334/ijic.8967.s4 Appendix 4. 8-step process for leading transformation towards IACOP close to home.

10.5334/ijic.8967.s5 Appendix 5. Strategies for trustworthiness and rigour of our findings.
