# Supplementary material for: Guiding Principles for Transformation Towards Integrated Acute Care for Older Adults Close to Home: Lessons from Nine Dutch Regional Partnerships. A Realist Evaluation
Source: Int J Integr Care. 2025 Jul 8;25(3):7. doi: 10.5334/ijic.8967 (PMC12247845; doi:10.5334/ijic.8967)
Supplement: Appendix 2. — RAMESES II and COREQ checklists. [file ijic-25-3-8967-s2.pdf]

We use RAMESES II reporting standards for realist evaluations. For the sake of readability, and considering author guidelines, information is sometimes provided in another section of the article than indicated in these reporting standards.

Table 1 List of items to be included when reporting realist evaluations

			Reported in document Y/N/Unclear	Page(s) in document
1		In the title, identify the document as a realist evaluation	Y	1
SUMMARY OR ABSTRACT				
2		Journal articles will usually require an abstract, while reports and other forms of publication will usually benefit from a short summary. The abstract or summary should include brief details on: the policy, programme or initiative under evaluation; programme setting; purpose of the evaluation; evaluation question(s) and/or objective(s); evaluation strategy; data collection, documentation and analysis methods; key findings and conclusions<br>Where journals require it and the nature of the study is appropriate, brief details of respondents to the evaluation and recruitment and sampling processes may also be included<br>Sufficient detail should be provided to identify that a realist approach was used and that realist programme theory was developed and/or refined	Y	2
INTRODUCTION				
3	Rationale for evaluation	Explain the purpose of the evaluation and the implications for its focus and design	Y	3
4	Programme theory	Describe the initial programme theory (or theories) that underpin the programme, policy or initiative	Y	3-5
5	Evaluation questions, objectives and focus	State the evaluation question(s) and specify the objectives for the evaluation.<br><br>Describe whether and how the programme theory was used to define the scope and focus of the evaluation	Y	2-5

			Reported in document Y/N/Unclear	Page(s) in document
6	Ethical approval	State whether the realist evaluation required and has gained ethical approval from the relevant authorities, providing details as appropriate. If ethical approval was deemed unnecessary, explain why	Y	6
METHODS				
7	Rationale for using realist evaluation	Explain why a realist evaluation approach was chosen and (if relevant) adapted	Y	4
8	Environment surrounding the evaluation	Describe the environment in which the evaluation took place	Y	7, appendix 1
9	Describe the programme policy, initiative or product evaluated	Provide relevant details on the programme, policy or initiative evaluated	Y	2
10	Describe and justify the evaluation design	A description and justification of the evaluation design (i.e. the account of what was planned, done and why) should be included, at least in summary form or as an appendix, in the document which presents the main findings. If this is not done, the omission should be justified and a reference or link to the evaluation design given. It may also be useful to publish or make freely available (e.g. online on a website) any original evaluation design document or protocol, where they exist	Y	7-9, 14

			Reported in document Y/N/Unclear	Page(s) in document
11	Data collection methods	Describe and justify the data collection methods – which ones were used, why and how they fed into developing, supporting, refuting or refining programme theory<br>Provide details of the steps taken to enhance the trustworthiness of data collection and documentation	Y	7-9,<br>14
12	Recruitment process and sampling strategy	Describe how respondents to the evaluation were recruited or engaged and how the sample contributed to the development, support, refutation or refinement of programme theory	Y	7-9
13	Data analysis	Describe in detail how data were analysed. This section should include information on the constructs that were identified, the process of analysis, how the programme theory was further developed, supported, refuted and refined, and (where relevant) how analysis changed as the evaluation unfolded	Y	8, 9
RESULTS				
14	Details of participants	Report (if applicable) who took part in the evaluation, the details of the data they provided and how the data was used to develop, support, refute or refine programme theory	Y	7-9
15	Main findings	Present the key findings, linking them to contexts, mechanisms and outcome configurations. Show how they were used to further develop, test or refine the programme theory	Y	9-13

DISCUSSION				
16	Summary of findings	Summarise the main findings with attention to the evaluation questions, purpose of the evaluation, programme theory and intended audience	Y	9, 13, appendix 3
17	Strengths, limitations and future directions	Discuss both the strengths of the evaluation and its limitations. These should include (but need not be limited to): (1) consideration of all the steps in the evaluation processes; and (2) comment on the adequacy, trustworthiness and value of the explanatory insights which emerged<br>In many evaluations, there will be an expectation to provide guidance on future directions for the programme, policy or initiative, its implementation and/or design. The particular implications arising from the realist nature of the findings should be reflected in these discussions	Y	13–15
18	Comparison with existing literature	Where appropriate, compare and contrast the evaluation's findings with the existing literature on similar programmes, policies or initiatives	Y	13, 14
19	Conclusion and recommendations	List the main conclusions that are justified by the analyses of the data. If appropriate, offer recommendations consistent with a realist approach	Y	14
20	Funding and conflict of interest	State the funding source (if any) for the evaluation, the role played by the funder (if any) and any conflicts of interests of the evaluators	Y	16

COREQ (COnsolidated criteria for REporting Qualitative research) Checklist

A checklist of items that should be included in reports of qualitative research. You must report the page number in your manuscript where you consider each of the items listed in this checklist. If you have not included this information, either revise your manuscript accordingly before submitting or note N/A.

Topic	Item No.	Guide Questions/Description	Reported on Page No.
**Domain 1: Research team and reflexivity**			
Personal characteristics			
Interviewer/facilitator	1	Which author/s conducted the interview or focus group?	8
Credentials	2	What were the researcher's credentials? E.g. PhD, MD	7
Occupation	3	What was their occupation at the time of the study?	7
Gender	4	Was the researcher male or female?	N/A
Experience and training	5	What experience or training did the researcher have?	7
Relationship with participants			
Relationship established	6	Was a relationship established prior to study commencement?	14
Participant knowledge of the interviewer	7	What did the participants know about the researcher? e.g. personal goals, reasons for doing the research	14
Interviewer characteristics	8	What characteristics were reported about the inter viewer/facilitator? e.g. Bias, assumptions, reasons and interests in the research topic	7,14
**Domain 2: Study design**			
Theoretical framework			
Methodological orientation and Theory	9	What methodological orientation was stated to underpin the study? e.g. grounded theory, discourse analysis, ethnography, phenomenology, content analysis	4,5
Participant selection			
Sampling	10	How were participants selected? e.g. purposive, convenience, consecutive, snowball	7
Method of approach	11	How were participants approached? e.g. face-to-face, telephone, mail, email	7
Sample size	12	How many participants were in the study?	7
Non-participation	13	How many people refused to participate or dropped out? Reasons?	8
Setting			
Setting of data collection	14	Where was the data collected? e.g. home, clinic, workplace	7,8
Presence of non-participants	15	Was anyone else present besides the participants and researchers?	7,8
Description of sample	16	What are the important characteristics of the sample? e.g. demographic data, date	Table 2
Data collection			
Interview guide	17	Were questions, prompts, guides provided by the authors? Was it pilot tested?	8
Repeat interviews	18	Were repeat inter views carried out? If yes, how many?	N/A.
Audio/visual recording	19	Did the research use audio or visual recording to collect the data?	8,9
Field notes	20	Were field notes made during and/or after the inter view or focus group?	No
Duration	21	What was the duration of the inter views or focus group?	8
Data saturation	22	Was data saturation discussed?	14
Transcripts returned	23	Were transcripts returned to participants for comment and/or	No

Topic	Item No.	Guide Questions/Description	Reported on Page No.
		correction?	
**Domain 3: analysis and findings**			
Data analysis			
Number of data coders	24	How many data coders coded the data?	8
Description of the coding tree	25	Did authors provide a description of the coding tree?	Appendix 3
Derivation of themes	26	Were themes identified in advance or derived from the data?	8
Software	27	What software, if applicable, was used to manage the data?	8
Participant checking	28	Did participants provide feedback on the findings?	8,9
Reporting			
Quotations presented	29	Were participant quotations presented to illustrate the themes/findings? Was each quotation identified? e.g. participant number	10-13
Data and findings consistent	30	Was there consistency between the data presented and the findings?	9-13
Clarity of major themes	31	Were major themes clearly presented in the findings?	9-13
Clarity of minor themes	32	Is there a description of diverse cases or discussion of minor themes?	14

Developed from: Tong A, Sainsbury P, Craig J. Consolidated criteria for reporting qualitative research (COREQ): a 32-item checklist for interviews and focus groups. *International Journal for Quality in Health Care*. 2007. Volume 19, Number 6: pp. 349 – 357

Once you have completed this checklist, please save a copy and upload it as part of your submission. DO NOT include this checklist as part of the main manuscript document. It must be uploaded as a separate file.
