# Supplementary material for: Guiding Principles for Transformation Towards Integrated Acute Care for Older Adults Close to Home: Lessons from Nine Dutch Regional Partnerships. A Realist Evaluation
Source: Int J Integr Care. 2025 Jul 8;25(3):7. doi: 10.5334/ijic.8967 (PMC12247845; doi:10.5334/ijic.8967)
Supplement: Appendix 3. — The program theory and coding tree. [file ijic-25-3-8967-s3.pdf]

Appendix 3: The program theory and coding tree with strategies, contexts, mechanisms and outcomes

Program theory

If (contexts)

the Guiding Principles for IACOP close to home

- Committing to a shared regional IACOP vision and goals (meso-level)
- Fostering a culture of collaborative and coordinated action (meso-level)
- Prioritising, implementing and developing interventions systematically (meso-level)
- Ensuring congruent health policy (macro-level)
- Stimulating functional integration (macro-level)
- Stimulating normative integration (macro-level)

… are applied in different functional and normative circumstances at the micro- meso- and macro-level

- Distribution of care (functional)
- Communication structure (functional)
- Funding (functional)
- Vision (normative)
- Culture (normative)

then (outcome)

stakeholder individuals and organizations are more likely to undertake ongoing action across integration levels, healthcare practices and sectors for transformation towards IACOP close to home *(outcome)*,

because (mechanisms)

1. they feel an increased sense of urgency;
2. there is more mutual understanding, and;
3. they enjoy the transformation process.

*once the primary mechanisms are set in motion, the secondary mechanisms can be triggered**:

4. a belief that transformation is necessary and beneficial;
5. a higher sense of population responsibility and ownership;
6. more trust between stakeholders and in the transformation process.

Table A3.1. Coding tree with strategies, contexts, mechanisms and outcomes

Strategies	Contexts	Mechanisms	Outcome
**Meso-level: Committing to a shared regional IACOP vision and goals**	**Micro level**	**Primary mechanisms**	**Positive impact on transformation towards IACOP close to home**
S: Creating a shared vision as regional partnership	C: Distribution of care (functional context)	M: Feeling of urgency	
S: Communicating the shared IACOP vision and goals continuously:	• C: Insight for professional in available regional care capacity	M: Mutual understanding	
• S: Communication by healthcare professionals with leadership qualities and/or roles	• C: Basic care for older person 'aging in place' lies with GP	M: Enjoying the process	
• S: Communication by directors and managers of healthcare organizations	• C: GP has certain autonomy regarding care provision (e.g. employing PAs)		
• S: Conveying the role of the concerned stakeholder(s) in the broader picture of IACOP close to home	• C: Overburdened healthcare professional(s) (e.g. GPs, district nurses)	**Secondary mechanisms**	**Negative impact on transformation towards IACOP close to home**
	• C: Shortage of specialists (elderly care physicians, geriatricians, NPs/PAs)	M: Belief that transformation is	
	• C: Wide range of choices in healthcare providers	necessary and beneficial	
**Meso-level: Fostering a culture of collaborative and coordinated action**	• C: Referral decision-making influenced by organizational factors (funding)	M: Sense of population responsibility	
S: Fostering collaborative and coordinated action between healthcare professionals by;	C: Communication structure (functional context)	and ownership	
• S: Having them work at the same (physical) location	• C: Frequency of interactions between professionals (from different practices)	M: Trust	
• S: Organizing joint courses for healthcare professionals from different organizations	• C: Awareness of the motives, strengths and weaknesses of other professionals		
• S: Providing joint training programs (f.e. internship both at hospital and nursing (home) care organization)	• C: Awareness of innovations		
• S: Implementing care models where care and cure are integrated (f.e. the AGCH)	• C: Staff turnover		
S: Fostering collaborative and coordinated action between organizations by;	• C: Use of different Electronic Health Records		
• S: (Re)structuring the regional partnership's governance	• C: Learning and improving based on data is applied in daily practice		
• S: Investing in the governance and organizational level of umbrella organizations for GP's,	• C: Patient communicates preferences (including Advance Care Planning)		
paramedics and pharmacists	• C: Expectation management between professional and patient		
	• C: Low literacy and/ or IT skills of patient		
	C: Funding (functional context)		
**Meso-level: Prioritizing, implementing and developing micro-level interventions systematically**	• C: Funding for professional for provided care is not sufficient (indirect time)		
S: Identifying shared bottlenecks for organizing IACOP close to home in the region;	• C: Difference in out-of-pocket costs when transitioning from ZVW to WLZ		
• S: Using quantitative (capacity) data	• C: Extent to which client/patient has utilized own risk deductible		
• S: Using practical experiences of healthcare professionals	C: Vision (normative context)		
• S: Mapping bottlenecks and improvement points along a patient journey map	• C: Professionals act (not) in accordance with the regional IACOP vision		
S: Prioritizing projects and interventions based on their expected impact on transformation towards IACOP;	• C: Professionals with leadership stimulate action for IACOP close to home		
• S: Alleviating the pressure on professionals in primary and district care	• C: Older adults aware of the regional IACOP vision (and urgency)		
• S: Implementing care models where cure and care are integrated (as catalysators for transformation)	C: Culture (normative context)		
• S: A shared wish (of stakeholder organizations) for implementation	• C: Habit to admit (complex) patients to ED (by e.g. GPs)		
S: Implementing and embedding interventions systematically through continuous learning and development;	• C: Divergent working cultures (e.g. hospital vs. long-term care)		
• S: Starting implementation with a select group of partners with (combined) clout	• C: Individual's creativity and perseverance		
• S: Signing as (implementation) partners a letter of intent or collaboration contract			
• S: Appointing an independent project leader (and/or team)	**Meso level**		
• S: Involving health insurers timely in the implementation process	C: Distribution of care (functional context)		
• S: Designing an implementation plan collectively, based on;	• C: Many/few nursing (home) providers (VVT)		
- The regional context, the estimated volume of patient/treatments, the available location(s), the deployable healthcare professionals, the desired presentation route(s).	• C: Presence/absence of geriatrics department		
	• C: Intermediate care department (STRC/GR) in hospital		
• S: Involving healthcare professionals timely in the implementation plan and process, by;	• C: Medical specialist companies (MSBs) / professionals on payroll		
- Assessing early the available and/or deployable healthcare professionals	• C: Renovation and/or new construction		
- Relieving healthcare professionals from project administration	• C: Capacity of intermediate care beds		
• S: Developing care pathways for the new care model timely	• C: 24/7 availability of intermediate care beds		
• S: Bringing the innovative care model continuously under the attention, using; - Protocols and care pathways, flowchart for referrals, appointing health care professional as ambassador, investing in connections with the core/stable group of healthcare professionals, communicating consistently the need of referrals and/or collaboration with the innovative care model.	• C: Many types of intermediate care beds		
	• C: 'Wrong bed problems' (i.e. admission to hospital without specialist indication)		
	• C: Joint capacity management		
	• C: Hospital care shifting towards primary care		
	C: Communication structure (functional context)		
• S: Organizing regional care coordination, by:	• C: Governance of regional partnership and/or umbrella organizations (for GPs)		
- Implementing regional care coordination centres, organizing tactical capacity meetings	• C: Changes in board and management		
between (managers) of organizations, appointing managers for care coordination	C: Funding (functional context)		
• S: Committing as stakeholders for IACOP close to home to a continuous improvement process	• C: Upfront investment costs		
- Through learning and improvement based on data at the professional level	• C: Low volume of treatment and/or patients for innovative care model		
- Through systematically (further) developing innovations at the organizational level	• C: Awareness of funding possibilities within existing structures		
	• C: Numerous procurement meetings with health purchasers		
	C: Vision (normative context)		
**Macro-level: Ensuring congruent health policy**	• C: Presence/absence clear regional vision		

S: Allocating decisive roles to national institution(s)	• C: Presence/absence clear organisational vision
S: Committing congruent behaviour between system organizations	• C: Conflicting interests and opinions
• S: Coordinating policy as national healthcare institutions (e.g. VWS, NZa, ZIN)	• C: Managers/executives stimulate action for IACOP close to home
• S: Adhering to the congruent procurement behaviour as health insurers	C: Culture (normative context)
S: Communicating development from management to operational staff within system organizations, such as;	• C: Market dynamics and ('managed') competition
• S: Communicating (up to date) information on innovative care models	• C: Sharing and discussing (conflicting) interests
• S: Communicating developments with regard to the regional context	• C: Prioritizing collective interests above individual and organizational interests
• S: Communicating relevant health policy developments	• C: Culture of adaptive innovation and learning within organization(s)
S: Providing generical care model (with core components) open to (context-based) customization	• C: Many (innovative) projects for IACOP close to home are initiated
**Macro-level: Stimulating functional integration**	**Macro level**
S: Aligning financial incentives with overarching system goals	C: Distribution of care (functional context)
• S: Letting go of strict business-case thinking	• C: Ageing populations
• S: Creating a financially secure environment (for hospitals) to enable shifts and integration of care	• C: Increasing multimorbidity
• S: Removing the p*q incentives from the system	• C: Tightening health labour market
• S: Adding incentives in the systems based on reducing overall patient trajectory costs	• C: Continuously changing healthcare system
• S: Providing integrated payment forms, such as:	• C: More ageing in place
– Intersectoral payment categories, bundled payments, population-based payment.	• C: Communication structure (functional context)
• S: Providing appropriate (conditions) for experimental financing	• C: Insight into available care capacity in the region
• S: Clarifying the process from experimental to structural financing for care providers	• C: Available legal and quality frameworks for integrated care and task shifting
• S: Providing appropriate funding for system functions	• C: Knowledge exchange on innovative care models
• S: Shifting focus from reducing costs to efficient utilization of health labour in the region	• C: Government-citizen communication structures
S: Creating (information) systems for regional care coordination and data exchange	C: Funding (functional context)
• S: Creating the right conditions for regional care coordination (centres)	• C: Siloed reimbursement system
• S: Stimulating easier and secure exchange of (care) capacity data	• C: Business-case thinking
• S: Stimulating easier and secure exchange of patient records	• C: Options for experimental funding
S: Providing legal and quality frameworks for integrated care	• C: Options for structural funding
• S: Providing legal frameworks to organizations who provide integrated care	• C: Funds for transformation
• S: Providing legal framework to professionals for task shifting	• C: P*Q (=production) incentives
• S: Providing quality frameworks for integrated care	• C: Rigid procurement frameworks
S: Applying a flexible approach for functional integration	• C: High turnover among purchasers
• S: Applying a flexible approach for financial integration	• C: Funding for system functions
• S: Providing (experimental) funding while (re)evaluating and enhancing effectiveness	C: Vision (normative context)
• S: Allowing customization of generic care models within (experimental) procurement frameworks, as long as the core components are maintained	• C: Policy documents with vision for future (acute) care (IZA, GALA, WOZO)<br>• C: The role of the nursing (home) care sectors in acute care is underexposed
	• C: Need for transformation towards IACOP is unclear and not communicated
**Macro-level guiding principle: Stimulating normative integration**	• C: Culture (normative context)
• S: Formulating the role of nursing (home) care in national policy (visions)	• C: Decisive roles are not communicated and/or taken
• S: Promoting a culture of innovative and experimental thinking	• C: No innovative thinking by system parties
• S: Managing citizen's expectations about future care	• C: Allowing context-based customization to (generic) care models
• S: Reframing and destigmatizing aging	• C: (Not) adhering to the congruent principle as health insurers
• S: Reframing aging from 'a sign of weakness' to a call for action to stimulate self-reliance	
• S: Destigmatizing aging amongst all citizens	
