# Supplementary material for: Guiding Principles for Transformation Towards Integrated Acute Care for Older Adults Close to Home: Lessons from Nine Dutch Regional Partnerships. A Realist Evaluation
Source: Int J Integr Care. 2025 Jul 8;25(3):7. doi: 10.5334/ijic.8967 (PMC12247845; doi:10.5334/ijic.8967)
Supplement: Appendix 4. — 8-step process for leading transformation towards IACOP close to home. [file ijic-25-3-8967-s4.pdf]

Appendix 4: 8-step process for leading transformation towards IACOP close to home

First create a climate for transformation;

1. Create sense of urgency: Transformation to IACOP close to home starts with a sense of urgency. This in line with a 'burning platform' as a trigger for system transformation [2]. Issues will be on the agenda of stakeholders when problems, such as care fragmentation, noncontinuity of care and/or low quality of care, become impossible to ignore.
2. Build a guiding coalition: A powerful regional coalition is needed to guide, coordinate and communicate the activities for IACOP close to home. If applicable, secure this coalition within the broader regional healthcare network.

Then commit to a shared regional IACOP vision with measurable goals;

3. Develop a clear shared vision and goals: The guiding coalition develops a compelling shared vision and clear measurable goals for transformation towards IACOP close to home. It is important that these address urgent issues to which patients and healthcare professionals can relate.
4. Communicate the vision and goals: Continuously communicate the vision to maintain urgency levels and to nurture the belief among stakeholders that transformation is not only necessary, but also beneficial. It's crucial to also convey the role of the concerned stakeholder(s) in the broader picture of transformation towards IACOP close to home. This triggers a sense of responsibility and ownership.

Next, foster a culture of collaborative and coordinated action;

5. Remove barriers: Identify and remove barriers for collaborative and coordinated action. Start as regional partnerships with barriers that impede mutual understanding and trust among stakeholders. Concurrently, system parties should undertake actions to remove barriers for joy and trust in the (transformation) process by promoting congruent health policy, functional and normative integration.

Then focus on prioritising, implementing and developing micro-level interventions systematically;

6. Create quick wins: Transformation towards IACOP close is a lengthy process. When this becomes clear to stakeholders, this might lead to a drop in urgency levels. Therefore, we recommend to first commit to quick wins when prioritizing, implementing and developing interventions for IACOP close to home. Celebrate quick wins to promote joy and trust in the transformation process.
7. Sustain momentum: After achieving some quick wins, transformation efforts can expand. Additionally, new initiatives can be launched that build on these first successes. To maintain joy and trust in the process, we advise to break-down complex and long-term projects into several smaller milestones and to celebrate each milestone reached. Concurrently, system parties should undertake actions to remove barriers for joy and trust in the (transformation) process by promoting congruent health policy, functional and normative integration
8. Institute change: Anchor new care models, interventions and/or behaviours in a culture of continuous learning and development for IACOP close to home. This will help to ensure that changes are sustained over the long-term, become part of daily operations and are further developed.

References appendix 4:

1. Kotter JP. Leading change. Boston: Harvard business press, 2012.
2. Moore J, Elliott IC, Hesselgreaves H. Collaborative leadership in integrated care systems; creating leadership for the common good. J. Chang. Manag. 2023 2;23(4):358-73.
