# Supplementary material for: Guiding Principles for Transformation Towards Integrated Acute Care for Older Adults Close to Home: Lessons from Nine Dutch Regional Partnerships. A Realist Evaluation
Source: Int J Integr Care. 2025 Jul 8;25(3):7. doi: 10.5334/ijic.8967 (PMC12247845; doi:10.5334/ijic.8967)
Supplement: Appendix 5. — Strategies for trustworthiness and rigour of our findings. [file ijic-25-3-8967-s5.pdf]

# Appendix 5: Strategies applied for trustworthiness and rigour of our findings

**Table A5.1.** Key four-dimension criteria strategies adapted from Lincoln and Guba [1]

| Rigor criteria | Purpose | Strategies applied |
|---|---|---|
| Credibility | To establish confidence that the results (from the perspective of the participants) are true, credible, and believable. | - We ensured that researchers had the required knowledge and research skills to perform their roles (e.g. qualitative and RE research training and peer consultation meetings).<br>- Member check of the guiding principles and underlying SCMO configurations by participants of the focus groups. |
| Dependability | To ensure the findings of this qualitative inquiry are repeatable if the inquiry occurred within the same cohort of participants, coders, and context. | - Rich description of the conceptual framework.<br>- Rich description of the study and the data collection and data analysis procedure.<br>- We developed a track record of the data collection.<br>- We tested for coding accuracy by spot checks (GH). |
| Confirmability | To extend the confidence that the results would be confirmed by other researchers. | - We applied several triangulation techniques (methodological, data source, investigators, theoretical).<br>- EK and GH held reflexive meetings to rearrange and rename the SCMO configurations.<br>- EK and AV held reflexive meetings after the focus groups to rearrange and rename the overarching SCMO configurations.<br>- The overarching SCMO configurations, clustered into guiding principles, were reviewed and confirmed with GH, SS, and BB. |
| Transferability | To extend the degree to which the results can be generalized or transferred to other contexts or settings. | - Purposeful sampling of study participants across nine regional partnerships.<br>- Applying a RE approach for 1) transferable guiding principles and mechanisms, and for 2) SCMO configurations with generalizable representations of which strategies work, for whom, when and in which circumstances. |

Reference:


1. Lincoln YS, Guba EG. But is it rigorous? Trustworthiness and authenticity in naturalistic evaluation. New Dir Progr Eval. 1986 Jun;1986(30):73-84.
